# Differences and associations of metabolic and vitamin D status among patients with and without sub-clinical hypothyroid dysfunction

**DOI:** 10.1186/1472-6823-13-31

**Published:** 2013-08-20

**Authors:** Naji J Aljohani, Nasser M Al-Daghri, Omar S Al-Attas, Majed S Alokail, Khalid M Alkhrafy, Abdulaziz Al-Othman, Sobhy Yakout, Abdulaziz F Alkabba, Ahmed S Al-Ghamdi, Mussa Almalki, Badurudeen Mahmood Buhary, Shaun Sabico

**Affiliations:** 1 Faculty of Medicine, King Saud bin Abdulaziz University for Health Sciences, King Fahad Medical City, Riyadh, Saudi Arabia; 2 Biochemistry Department, Biomarkers Research Program, College of Science, King Saud University, Riyadh, Saudi Arabia; 3 Prince Mutaib Chair for Biomarkers of Osteoporosis, King Saud University, Riyadh, Saudi Arabia; 4 Center of Excellence in Biotechnology Research Center, King Saud University, Riyadh, Saudi Arabia; 5 Clinical Pharmacy Department, College of Pharmacy, King Saud University, Riyadh, Saudi Arabia; 6 College of Applied Medical Sciences, King Saud University, Riyadh, KSA, Saudi Arabia

**Keywords:** Thyroid dysfunction, Obesity, Dyslipidemia, Saudi

## Abstract

**Background:**

Sub-clinical hypothyroid dysfunction, a relatively understudied disorder in the Kingdom of Saudi Arabia (KSA), has significant clinical implications if not properly monitored. Also from KSA, more than 50% of the population suffer from hypovitaminosis D (<50 nmol/l). In this cross-sectional case-control study, we described the differences and associations in the metabolic patterns of adult Saudis with and without hypothyroid dysfunction in relation to their vitamin D status, PTH, calcium and lipid profile.

**Methods:**

A total of 94 consenting adult Saudis [52 controls (without subclinical hypothyroidism), 42 cases (previously diagnosed subjects)] were included in this cross-sectional study. Anthropometrics were obtained and fasting blood samples were taken for ascertaining lipid and thyroid profile, as well as measuring PTH, 25(OH) vitamin D and calcium.

**Results:**

Cases had a significantly higher body mass index than the controls (*p* < 0.001). Circulating triglycerides was also significantly higher in cases than the controls (*p* = 0.001). A significant positive association between HDL-cholesterol and PTH (R = 0.56; *p* = 0.001), as well as a negative and modestly significant negative association between LDL-cholesterol and PTH (R = - 20.0; *p* = 0.04) were observed. FT3 was inversely associated with circulating 25 (OH) vitamin D (R = -0.25; *p* = 0.01).

**Conclusions:**

Patients with hypothyroid dysfunction possess several cardiometabolic risk factors that include obesity and dyslipidemia. The association between PTH and cholesterol levels as well as the inverse association between vitamin D status and FT3 needs to be reassessed prospectively on a larger scale to confirm these findings.

## Background

Thyroid diseases are one of the more commonly encountered endocrine disorders in the Kingdom of Saudi Arabia (KSA), next to diabetes mellitus (DM) [1]. While current demographics point to less alarming prevalence of thyroid dysfunction, certain populations such as children with Down syndrome appear to manifest conditions related to thyroid dysfunction [2]. In the Middle East in general, thyroid dysfunction, specifically subclinical hypothyroid dysfunction, is a relatively understudied field as compared to other metabolic disorders such as obesity and insulin resistance. In a recent study, Bahammam and colleagues observed that subclinical hypothyroid dysfunction was prevalent among Saudi patients suffering from obstructive sleep apnea (OSA), with an estimated 1 out of 10 OSA patients harboring the condition [3]. Granted that hypothyroid dysfunction is not as endemic as the previously mentioned disorders, it nevertheless deserves attention as it can lead to serious repercussions if left ignored. Several studies point to an association between thyroid diseases, particularly Grave’s disease and vitamin D status in several ethnic groups, but none if not limited in the Middle East [4-6]. In this cross-sectional case-control study, we describe the differences and associations in the metabolic patterns of adult Saudis with and without subclinical hypothyroid dysfunction in relation to lipid and calcium metabolism in an attempt to stimulate further clinical investigations with respect to this understudied disorder.

## Methods

### Subjects

A total of 94 consenting adult Saudis [52 control (6 males; 46 females), 42 cases (3 males, 39 females)] were included in this cross-sectional study. All subjects were recruited at the Endocrinology Unit of King Fahad Medical City, Riyadh Saudi Arabia. Adult Saudis aged 20-50, who are known cases of subclinical hypothyroid dysfunction based on previous clinical assessment (elevated TSH with normal FT4 levels), medications taken and laboratory tests with no complications were included. Control subjects were those who tested negative for subclinical hypothyroid dysfunction with no history of thyroid medications. Non-Saudis and pregnant women as well as children were excluded. Written and informed consent were secured prior to inclusion. Ethical approval was obtained from the Institutional Review Board of King Fahad Medical City, Riyadh, KSA.

### Anthropometrics

Anthropometric parameters were obtained while the subject was standing erect and barefoot. Height and weight were determined using standardized conventional methods. Body mass index (BMI) was calculated using the formula: weight in kilograms (kg) divided by height in squared meters (m^2^).

### Blood measurements

Blood (≈ 10cc) was withdrawn after an overnight fast (> 10 hours). Serum calcium was measured using standard analytical techniques (Konelab, Finland). Fasting blood lipids which included triglycerides, total, HDL- and LDL-cholesterol as well as calcium were measured routinely using a chemical analyzer (Konelab, Finland). Serum TSH, FT3, and FT4 were estimated using commercially available kits by Roche Elecsys Modular Analytics Cobas e411 utilizing electrochemiluminescence immunoassay (Roche Diagnostics, Mannheim, Germany). TSH upper normal limit for males is 3.69 μIU/ml and 3.94 μIU/ml for females. Intact PTH and 25(OH)D were measured by specific ELISAs in accordance with the instructions provided by the manufacturer (IDS, Tyne & Wear, UK). 25(OH) vitamin D has been the preferred metabolite for the measurement of vitamin D status instead of 1, 25(OH)D, and remains the basis for the diagnosis of vitamin D deficiency [7]. The inter- and intra-assay variabilities were 5.8% and 3.4% respectively for the intact PTH ELISA, 5.3% and 4.6% respectively for the 25(OH)D ELISA. Anti-thyroid peroxidase (TPO) antibodies (Ab) were also measured using commercially available ELISA kits (Bio-Line S.A, Brussels, Belgium) with a sensitivity of 1.4 U/ml (intra- assay variability 6.9%; inter-assay variability 13.4%).

### Data analysis

Data was analyzed using the Statistical Package for the Social Sciences (SPSS) version 16.5 (Chicago, IL, USA). Frequencies were presented as N and continuous variables if normal were presented as mean ± standard deviation and median (inter-quartile range) for variables with non-normal distribution. For group comparisons (controls versus cases), independent T-test if normally distributed continuous variables, and Mann-Whitney U-test for variables with non-Gaussian distribution were utilized. Significance set at *p* < 0.05.

## Results

**Figure 1:**
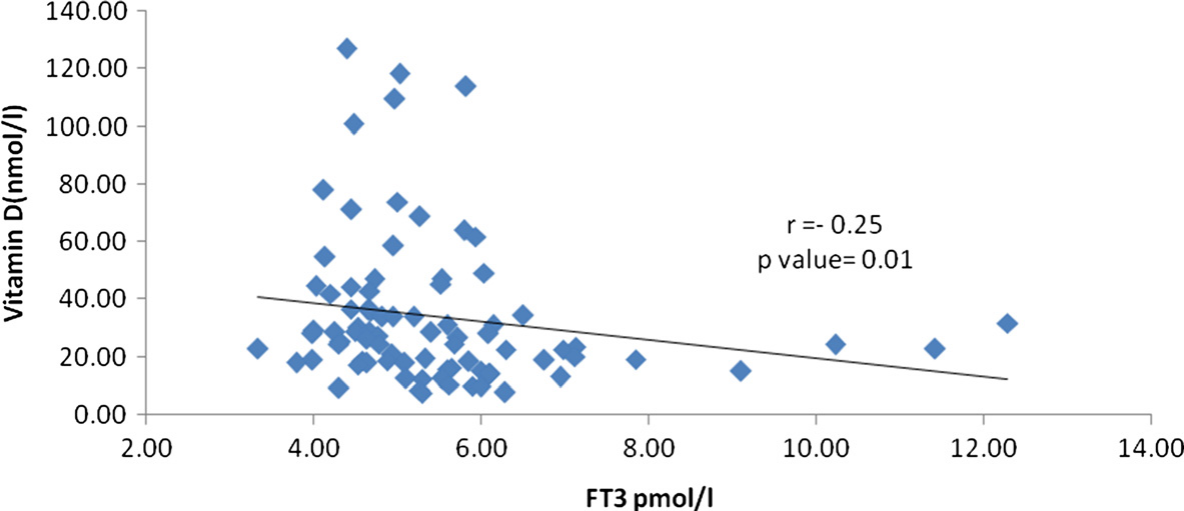
Inverse association between FT3 and 25(OH) Vitamin D.

Table 1 shows the general characteristics of both cases and controls. There was no significant difference in the mean age of both groups. Subjects with thyroid dysfunction had a significantly higher body mass index than controls (*p* < 0.001). Circulating triglycerides were also significantly higher among the cases than the controls (*p* = 0.001). The rest of the lipid parameters (Total, LDL- and HDL-cholesterol) were not significant. Among the thyroid tests, median TSH and median TPO Ab were significantly higher in the cases (consistent with Hashimoto’s Thyroiditis) who also had a significantly lower mean FT3 and iodine than the controls (*p* values 0.03, 0.001, 0.009 and < 0.001, respectively). Only 2 subjects had TPOAb levels below 1.7 U/ml (cases). Mean FT4 was similar in both cases and controls. Lastly, mean serum calcium as well as corrected calcium were significantly lower in cases than controls (*p* = 0.002 and *p* ≤ 0.001, respectively). Cases also had a significantly higher 25 (OH) vitamin D levels than the controls (*p* < 0.001), although both groups fall below sufficiency levels. Intact PTH was not significantly different between groups. Table 2 shows the regression coefficients of the lipid parameters versus the different thyroid tests, as well as 25(OH) vitamin D and PTH. A significant positive association between HDL-cholesterol and PTH (R = 0.56; *p* = 0.001) as well as a negative and modestly significant association between LDL-cholesterol and PTH were detected (R = - 20.0; *p* = 0.04). The rest of the associations were not significant. Finally, Figure 1 shows the inverse and significant association between FT3 and circulating 25 (OH) vitamin D (R = -0.25; *p* = 0.01). TSH was not significantly associated with 25(OH) vitamin D.

**Table 1:** Differences in metabolic characteristics of controls versus cases

	**Controls**	**Cases**	**P value**
N	52	42	
Age (years)	36.1 ± 8.1	35.5 ± 10.1	0.75
Gender (M/F)	6/46	3/39	
Body Mass Index (kg/m^2^)	27.8 ± 3.9	32.3 ± 6.8	<0.001
Triglycerides (mmol/l)	1.2 ± 0.26	1.9 ± 0.46	0.001
Total Cholesterol (mmol/l)	5.1 ± 0.80	5.2 ± 1.0	0.56
LDL-Cholesterol (mmol/l)	3.9 ± 0.88	3.9 ± 0.87	0.99
HDL-Cholesterol (mmol/l)	0.90 ± 0.37	0.88 ± 0.30	0.78
Iodine (μg/l)	119.5 ± 35.9	97.6 ± 38.5	0.009
TSH (mIU/l)#	2.4 (1.6, 3.3)	3.7 (1.5, 7.6)	0.04
FT3 (pmol/l)	6.0 ± 1.3	4.8 ± 1.2	<0.001
FT4 (pmol/l)	17.2 ± 1.3	17.5 ± 1.2	0.80
TPOAb (U/ml) #	17.7 (14.1, 25.0)	164.9 (15.8, 582.9)	0.001
PTH (pg/ml)	5.3 ± 1.5	8.0 ± 1.2	0.15
Albumin (g/l)	33.8 ± 5.4	36.5 ± 6.8	0.03
Corrected Ca (mmol/l)	2.4 ± 0.23	2.1 ± 0.18	< 0.001
Ca (mmol/l)	2.5 ± 0.19	2.1 ± 0.45	0.002
25 (OH) Vitamin D (nmol/l)	18.3 ± 1.5	37.3 ± 1.9	< 0.001

**Note**: # denotes non-Gaussian distribution and presented as median (interquartile range); *p*-value significant at < 0.05.

**Table 2:** Correlation coefficients (R) of Lipids to thyroid function tests, PTH and vitamin D adjusted for age and BMI

	**TSH**	**FT3**	**FT4**	**PTH**	**Iodine**	**TPO Ab**	**Vitamin D**
Total Cholesterol (mmol/l)	-0.05	-0.02	-0.06	-0.04	0.05	0.03	-0.02
HDL-Cholesterol (mmol/l)	-0.12	-0.13	-0.13	0.56**	-0.04	0.08	-0.04
Triglycerides (mmol/l)	0.12	0.04	-0.10	-0.16	-0.09	0.01	-0.19
LDL-Cholesterol (mmol/l)	-0.04	0.02	0.003	-0.20*	0.08	0.001	0.04

**Note**: * denotes significance at < 0.05; ** denotes significance at < 0.01; *p*-value significant at < 0.05.

## Discussion

The main finding of this study is that aside from the conventional and expected differences in the expression of thyroid tests, including iodine and TPOab, patients with hypothyroid dysfunction also exhibited obesity and elevated levels of triglycerides as compared to controls. How subclinical hypothyroid dysfunction influences weight gain can be explained via the peripheral effects of thyroid hormones and their local regulation of central nervous system (CNS) in the physiologic regulation of appetite that is independent of their conventional role in basal energy expenditure as well as regulation of resting energy expenditure [8,9]. With respect to obesity per se, it has been found out that visceral adipose tissue has a direct effect in TSH levels among obese, and this is independent of insulin resistance [10]. Regarding triglycerides, our results were in accordance with the study of Wanjia and colleagues, who also observed elevated TSH levels among patients with hypertriglyceridemia [11]. The exact mechanisms accountable for the effects of thyroid function with respect to lipid profile are unclear. Nevertheless it is almost established that TSH receptors are present in tissues other than the thyroid gland which include kidneys, bone marrow and adipose tissue, and that variations ion TSH levels among euthyroid patients are partially explained by lipid components and hypercholesterolemia which are independent of thyroid hormones [12,13]. Furthermore, there are several factors not accounted for in this study that may influence thyroid function and lipid profile, including smoking and insulin resistance [14], as well as obesity and abnormalities in glucose tolerance.

One striking and unexpected finding in this study is the significantly higher mean 25(OH) vitamin D levels among patients with subclinical hypothyroid dysfunction than controls despite the presence of established risk factors for vitamin D deficiency including obesity and hypetriglyceridemia [15-17]. Calcium levels while also significantly different, still fall within normal range, although the possibility of other causes such as malabsorption or decreased dietary calcium intake in some subjects cannot be ruled out. Furthermore, PTH levels were higher, although not significant in the case group than controls. These differences this can be attributed to several confounders unaccounted for in the study which include differences in dietary calcium and vitamin D intake, undocumented comorbidities such as malabsorption and existing renal disorders which were not evident at the time of selection, as well as sun exposure, and needs further investigation. Nevertheless, while the vitamin D status of cases was significantly higher as compared to controls, both mean levels were still far below sufficiency levels and the corresponding PTH levels were not significantly different from one another, and as such the interpretation is comparable. Furthermore we recently observed that PTH levels do not correlate with 25(OH) vitamin D levels in Saudis despite severely low levels [18].

The association of PTH with lipid concentrations, specifically the cholesterol levels was demonstrated earlier and we suggest that this is mainly due to the positive association of PTH to body composition and adiposity, rather than the cholesterol itself [19,20]. Also, the inverse association of FT3 to circulating levels of 25(OH)D should be interpreted with caution. It was previously reported that higher vitamin D status was associated with low TSH only among younger individuals and not in adults [21]. Furthermore, while vitamin D deficiency has been implicated in several autoimmune thyroid diseases, no association was elicited between the antibodies measured in the present study and vitamin D, confirming a recent study among Dutch natives about the lack of correlation of low vitamin D levels and the early stages of thyroid auto-immunity [22]. Similarly, polymorphisms of vitamin D alpha-hydroxylase (CYP)1alpha, a key enzyme for regulating both systemic and tissue levels of 1, 25-dihydroxyvitamin D3 [23], did not correlate with known auto-immune disorders such as type 1 diabetes mellitus, Grave’s disease and Hashimoto’s thyroidits among Caucasian pedigrees [24]. In this context, it should be taken into account that TSH may have direct and independent effects on bone metabolism regardless of thyroid hormones [25,26].

The authors acknowledge several limitations. The cross-sectional nature of the study and the small sample size limits the findings of the study to at best, suggestive. Several major confounders were also not included including season which has a counterintuitive effect in the vitamin D status of citizens residing in the Gulf region [27]. Gender difference could not be elicited due to the big discrepancy in the numbers between male and female subjects. Nevertheless, the study has its own strength as being the first to document several metabolic differences among Saudi patients with and without hypothyroid dysfunction and the first to associate vitamin D status in relation to thyroid function profile.

## Conclusions

In summary, adult Saudi patients with subclinical hypothyroid dysfunction harbor several cardiometabolic abnormalities, including obesity and dyslipidemia, the latter being associated positively with PTH levels. Higher vitamin D levels were associated with lower FT3 in the patents. Further prospective studies are needed to determine whether the associations elicited are causal to one another.

## Competing interests

The authors declare that they have no competing interest.

## Authors’ contributions

NJA and NMA conceived the study. OSA, MSA, KMA and AA carried out data acquisition and interpretation. SY, AFA, ASA, MA and BMB analyzed the data and prepared the manuscript. NJA and SS drafted the revised and final version of the manuscript. All authors provided intellectual contributions to the manuscript and has read and approved the final version.

## Pre-publication history

The pre-publication history for this paper can be accessed here:

http://www.biomedcentral.com/1472-6823/13/31/prepub
